# Salinity-Mediated Increment in Sulfate Reduction, Biofilm Formation, and Quorum Sensing: A Potential Connection Between Quorum Sensing and Sulfate Reduction?

**DOI:** 10.3389/fmicb.2019.00188

**Published:** 2019-02-06

**Authors:** Krishnakumar Sivakumar, Giantommaso Scarascia, Noor Zaouri, Tiannyu Wang, Anna H. Kaksonen, Pei-Ying Hong

**Affiliations:** ^1^Water Desalination and Reuse Center, Biological and Environmental Sciences and Engineering Division, King Abdullah University of Science and Technology, Thuwal, Saudi Arabia; ^2^Land and Water, Commonwealth Scientific and Industrial Research Organization, Floreat, WA, Australia

**Keywords:** salinity, biological sulfate reduction, biocorrosion, *Desulfovibrio vulgaris*, *Desulfobacterium corrodens*, quorum sensing inhibitors

## Abstract

Biocorrosion in marine environment is often associated with biofilms of sulfate reducing bacteria (SRB). However, not much information is available on the mechanism underlying exacerbated rates of SRB-mediated biocorrosion under saline conditions. Using *Desulfovibrio* (*D.*) *vulgaris* and *Desulfobacterium* (*Db.*) *corrodens* as model SRBs, the enhancement effects of salinity on sulfate reduction, *N*-acyl homoserine lactone (AHL) production and biofilm formation by SRBs were demonstrated. Under saline conditions, *D. vulgaris* and *Db. corrodens* exhibited significantly higher specific sulfate reduction and specific AHL production rates as well as elevated rates of biofilm formation compared to freshwater medium. Salinity-induced enhancement traits were also confirmed at transcript level through reverse transcription quantitative polymerase chain reaction (RT-qPCR) approach, which showed salinity-influenced increase in the expression of genes associated with carbon metabolism, sulfate reduction, biofilm formation and histidine kinase signal transduction. In addition, by deploying quorum sensing (QS) inhibitors, a potential connection between sulfate reduction and AHL production under saline conditions was demonstrated, which is most significant during early stages of sulfate metabolism. The findings collectively revealed the interconnection between QS, sulfate reduction and biofilm formation among SRBs, and implied the potential of deploying quorum quenching approaches to control SRB-based biocorrosion in saline conditions.

## Introduction

Limited availability of freshwater has led to the use of seawater in several industrial applications. High chloride and sulfate content in seawater coupled with biochemical reactions mediated by microorganisms accelerates the rate of biocorrosion in marine environments. Among these microorganisms, sulfate reducing bacteria (SRB) play a crucial role in biocorrosion and biofouling through biofilm formation, hydrogen sulfide production and extracellular electron transfer (Beech et al., 2005; Kuang et al., 2007; Zhang et al., 2011; Kato, 2016; Scarascia et al., 2016).

Biocorrosion in marine environment has often been associated with SRB biofilms (Beech and Sunner, 2004). In *Desulfovibrio vulgaris* (an SRB) biofilm-associated cells, upregulation of hydrogenases and cytochrome *c*533, both of which act as electron conduits, suggest the role of SRB biofilms in microbial-induced corrosion (Pereira et al., 2011; Clark et al., 2012; Scarascia et al., 2016). Recent genomic studies have shown that *D. vulgaris* biofilm-associated cells often exhibit high levels of gene expression heterogeneity related to exopolysaccharide synthesis, histidine kinases involved in biofilm formation as well as hydrogenases and cytochromes (Zhang et al., 2007; Caffrey et al., 2008; Krumholz et al., 2015; Qi et al., 2016). Earlier studies have also reported on induction of putative formate dehydrogenases and Ech hydrogenases under saline conditions (Mukhopadhyay et al., 2006; Clark et al., 2012). Another study found that high levels of salinity (35 g/L NaCl) did not compromise the metabolic activity of carbon steel-associated SRB biofilms, which in turn exacerbated the rate of biocorrosion (De França et al., 2000). Taken together, it is hypothesized that salinity accelerates biocorrosion by inducing SRB-mediated biofilm formation and sulfate reduction at the gene expression level.

Earlier studies have already established the correlation between biofilm formation and quorum sensing (QS) (Davies et al., 1998; Hammer and Bassler, 2003; Parsek and Greenberg, 2005). It is therefore hypothesized that the increase in SRB biofilm formation and sulfate reduction in the saline environment would be associated with QS mechanisms. Previous studies have reported on the production of QS signal molecules such as *N*-acyl homoserine lactones (AHLs) [*N*-hexanoyl-homoserine lactone (C6-HSL) to *N*-dodecanoyl-homoserine lactone (C12-HSL)] by SRB species (Decho et al., 2009, 2010). Compared to other bacterial species such as *Vibrio* sp. and *Pseudomonas* sp., relatively little information is available on QS in SRB.

Extensive genomic mining of *Desulfovibrio* species mainly revealed the presence of proteins homologous to putative QS receptor proteins such as LuxR. However, since synthases were not discovered from genomic mining of SRBs, SRB-based LuxR proteins may be simply orphan receptors and hence, may or may not be involved in QS (Scarascia et al., 2016). Comprehensive genomic analysis of *Desulfovibrio* species has also revealed the presence of several two-component signal transduction systems, whose exact function in SRB biofilm formation is relatively unknown (Kawaguchi et al., 2008; Decho et al., 2010; Scarascia et al., 2016). It has been speculated that sensor histidine kinases, which dominate these signal transduction systems might be linked with cell–cell communication within SRB biofilms (Zhang et al., 2007; Rajeev et al., 2011). Hence, the exact mechanism of QS in SRBs as well as its linkage to sulfate reduction is largely unknown and it would be interesting to investigate the connection between QS, sulfate reduction and biofilm formation by SRBs under saline conditions.

To explore the connection between QS, sulfate reduction and biofilm formation by SRBs under saline conditions, *Desulfovibrio* (*D.*) *vulgaris* Hildenborough and *Desulfobacterium* (*Db*.) *corrodens* were used as model SRBs in this study. *D. vulgaris* is a well-studied SRB with its entire genome sequenced and annotated, whereas *Db. corrodens* is a highly corrosive SRB well suited to iron-rich environments, whose genome has been annotated but with no evidence on the presence of QS-based gene homologs (Bryant et al., 1977; Dinh et al., 2004; Heidelberg et al., 2004; Clark et al., 2007; Gittel et al., 2010). Both species were propagated in either saline or freshwater media in the presence of lactate and Na_2_SO_4_ as electron donor and acceptor, respectively. Enhanced rates of sulfate reduction, AHL production and biofilm formation by *D. vulgaris* and *Db. corrodens* were observed under saline conditions. To further understand the influence of salinity on SRB at the gene expression level, we quantified transcript levels of genes related to sulfate reduction, carbon utilization, biofilm formation-based hydrogenases and cytochromes as well as histidine kinases involved in cell–cell communication. The results demonstrated that transcript levels of all selected genes were significantly upregulated under saline conditions. Hence, salinity has a pronounced effect on sulfate reduction, biofilm formation and AHL production at genetic level by both planktonic cells and biofilms of SRB. Further, by deploying QS inhibitors, it was demonstrated that the correlation between QS and sulfate reduction displayed by SRBs is most significant during early stages of sulfate metabolism. The findings suggest that QSI could be deployed as potential biocides to inhibit SRB biofilm-mediated biocorrosion during the early phases of biofilm formation but not on mature SRB biofilm.

## Materials and Methods

### Bacterial Strains, Media, and Culture Conditions

*Desulfovibrio vulgaris* Hildenborough (Heidelberg et al., 2004) and *Desulfobacterium corrodens* (DSM 15630) were propagated in either saline or freshwater media recommended by Leibniz Institute DSMZ, German Collection of Microorganisms and Cell Cultures. *D. vulgaris* strain used in this study harbors its 200 kbp native plasmid pDV1, whose presence has been reported to be crucial in its biofilm formation and maintenance (Clark et al., 2007). Saline medium (modified DSMZ medium 141) had the following composition (concentration in g/L) (salinity = 25.9g/L): KCl, 0.34; MgCl_2_.6H_2_O, 4.00; NH_4_Cl, 0.25; CaCl_2_.2H_2_O, 0.14; K_2_HPO_4_, 0.14; NaCl, 20; yeast extract, 1; tryptone, 1. Dissolved ingredients were initially autoclaved and then supplemented with 5 g/L NaHCO_3_ and 10 mL/L of DSMZ-141 vitamin solution (10×) and DSMZ-141 trace element solution (10×) from their respective filter-sterilized stock solutions (Bajracharya et al., 2015, 2017). Freshwater medium (modified DSMZ 641) had the following composition (concentration in g/L) (salinity = 4.17 g/L): MgCl_2_, 2; K_2_HPO_4_, 0.50; NH_4_Cl, 1; CaCl_2_, 0.75; yeast extract, 1; tryptone, 1. The freshwater medium was autoclaved and further supplemented with 5 g/L NaHCO_3_, 10 mL/L of DSMZ-141 vitamin solution (10×) and 1 mL/L of SL-10 trace element solution (10×) (from DSMZ medium 503) (Kádár et al., 2003; Ünal et al., 2012). Sodium lactate and Na_2_SO_4_ at final respective concentrations of 20 mM (2.24 g/L) and 10 mM (1.42 g/L) were added to both media to serve as electron donor and acceptor, respectively (Bryant et al., 1977; McInerney and Bryant, 1981; Krumholz et al., 2015). The pH of the saline and freshwater media was adjusted to 7.30, and both media were filtered through 0.25 μm syringe filter prior transferring to sterile autoclaved anaerobic tubes. All the tubes were sealed with butyl rubber stoppers and then maintained under anaerobic environment by purging the media with N_2_ for 10 min. Further, 0.50 g/L Na_2_S.9H_2_O was added to saline and freshwater media inside anaerobic chamber (Coy Laboratory Products Inc., Grass Lake, MI, United States). All cultures were incubated at 30°C on a rotary shaker.

### Sulfate Analysis

Sulfate concentration in extracted samples (diluted 100×) were quantified using a Dionex ICS-1600 Ion Chromatography system (Dionex Corp., Sunnyvale, CA, United States) equipped with a high-pressure pump, a sample auto-injector, a guard and separator column, a chemical suppressor, a conductivity cell and a data collection system with KOH as the eluent. Data collection and processing were regulated by software Chromeleon 7.0 (Dionex Corp., Sunnyvale, CA, United States) (Altland and Locke, 2012; Uzhel et al., 2016).

### Quantification of Cell Density

Cell density of *D. vulgaris* and *Db. corrodens* propagated in saline and freshwater media were measured using Accuri C6 Flow cytometry system (BD Bioscience, Franklin Lakes, NJ, United States) using protocols described earlier (Cheng et al., 2016). Cell pellets harvested through centrifugation (12,000 × *g*, 15 min) was washed (two times) with 0.9% NaCl. Prior to flow cytometry, diluted cell suspensions (10^5^–10^6^ cells/mL) were stained with SYBR green (Invitrogen AG, Bazel, Switzerland), diluted 10^4^-times from stock concentration (10^4^-fold concentrated in DMSO) (Marie et al., 1997; Noble and Fuhrman, 1998). After staining, cells were incubated at room temperature under dark conditions for 15 min. About 50 μL of stained cells were injected at a flow rate of 35 μL/min to Accuri C6 Flow cytometry system and then excited at 488 nm to enumerate the cell density. In order to evaluate differences in morphological changes between saline and freshwater media, black spots within the flow cytometry gating region were observed. No significant change was observed, which suggested no change in morphology between saline and freshwater media.

### Extraction and Quantification of Total *N*-Acyl Homoserine Lactones

For extraction of total AHLs, cell-free extracts of both SRB grown in saline and freshwater media were collected by centrifugation (12,000 × *g*, 15 min). Cell-free extracts were re-concentrated initially through lyophilization and then by resuspending the lyophilized fraction to 1/10th of the initial extracted volume in autoclaved H_2_O (pH 6.7). Total AHLs were quantitatively determined using a bioassay with beta-glo (Promega, United States) as luminescence substrate (Kawaguchi et al., 2008; Decho et al., 2009). Bioluminescence assay was conducted using a flat white 96-well plate (Greiner Bio-One, Sigma-Aldrich, MI, United States). Briefly, 20 μL of samples were mixed with 80 μL of *Agrobacterium* (*A.*) *tumefaciens* NT1 biosensor prepared in AT medium (Kawaguchi et al., 2008). After incubation for 90 min at 30°C, 100 μL of Beta-Glo reagent were added into each well of the 96-well plate. After incubation for 30 min at room temperature, bioluminescence intensity of each sample was recorded using a microplate reader (TECAN M200, M200, Männedorf, Switzerland) (Kawaguchi et al., 2008; Decho et al., 2009). Biosensor *A. tumefaciens* NT1 harbors ß-galactosidase enzyme, whose expression is regulated by the presence of AHLs. Beta-galactosidase cleaves beta-glo substrate to form luciferin in the presence of AHLs, which generates luminescence (Kawaguchi et al., 2008). Each bioassay was conducted in triplicate to assess reproducibility. Different AHLs such as *N*-butanoyl-homoserine lactone (C4-HSL), *N*-hexanoyl-homoserine lactone (C6-HSL), *N*-octanoyl-homoserine lactone (C8-HSL), *N*-decanoyl-homoserine lactone (C10-HSL), *N*-dodecanoyl-homoserine lactone (C12-HSL), *N*-tetradecanoyl homoserine lactone (C14-HSL), *N*-hexadecanoyl homoserine lactone (C16-HSL) and *N*-octadecanoyl homoserine lactone (C18-HSL) were used to prepare standard curves to optimize the bioluminescence assay (Supplementary Figure S1A). Dominant AHLs produced by SRBs were analyzed using liquid chromatography (LC) – mass spectrometry (MS)/MS (Agilent Technologies, Santa Clara, CA, United States) using protocols described elsewhere (Ortori et al., 2011) (Supplementary Figure S1B). For LC-MS/MS analysis, a part of cell-free extract was mixed with equal volumes of dichloromethane. AHL extraction procedure was repeated three times, and the organic solvent was evaporated to complete dryness using anhydrous Na_2_SO_4_. Dried samples were re-dissolved in methanol prior to analysis (McClean et al., 1997).

### Effect of Salinity on Sulfate Reduction and AHL Production

To elucidate effects of salinity on planktonic cells, *D. vulgaris* and *Db. corrodens* were propagated in saline and freshwater media using lactate and Na_2_SO_4_ as electron donor and electron acceptor respectively. Test conditions and media composition were as described earlier. A working volume of 22 mL was maintained in each anaerobic tube. Three biological replicates were used for each test conditions. About 1 mL of culture was extracted from anaerobic tubes every 24 h, and then centrifuged at 12,000 × *g* for 15 min. Harvested cell pellets were used to enumerate cell density with flow cytometry (Cheng et al., 2016). Cell-free supernatant was used to quantify sulfate concentration (100× diluted) and total AHLs produced, corresponding to each time interval. The effects of salinity were quantified in terms of specific sulfate reduction rate and specific AHL production rate (Fründ and Cohen, 1992; Detmers et al., 2001; Flodgaard et al., 2003; Bruhn et al., 2004). Specific sulfate reduction rate was defined in terms of total amount (μmoles) of sulfate reduced per cell per unit time (μmoles of sulfate/cell/h), whereas specific AHL production rate was expressed in terms of the total amount (nmoles) of AHLs synthesized per cells per unit time (nmoles of AHLs/cell/h).

### Effect of Salinity on Biofilm Formation

To elucidate effects of salinity on SRB biofilm formation, a static biofilm assay was conducted on *D. vulgaris* and *Db. corrodens* biofilms cultivated on a polystyrene flat bottom 96-well plate (Costar, Corning Inc., Corning, NY, United States). A total of eight biological replicates, with three technical replicates per each biological replicate, were used for this study. The biofilms were propagated using both saline and freshwater media. Biofilms were incubated at 30°C for 144 h within anaerobic chamber (Coy Laboratory Products Inc., Grass Lake, MI, United States). After 8 days, planktonic cells were removed and cells attached to the bottom of wells were washed with sterile 0.9% NaCl. Attached cells were then stained with 100 μL of 1% crystal violet (CV) reagent. After staining, cells were incubated at room temperature for 15 min. Excess CV was removed from each well, which was then air dried. Attached cells were then resuspended in 100 μL of 96% ethanol. Biofilm biomass was quantified in terms of OD_590_ using a microplate reader (SpectraMax 340PC384, Molecular Devices, CA, United States).

### Reverse Transcription Quantitative Polymerase Chain Reaction (RT-qPCR)

Reverse transcription quantitative polymerase chain reaction (RT-qPCR)-based approach was selected to quantify the expression of target genes associated with sulfate utilization, carbon and energy metabolism as well as biofilm formation in *D. vulgaris*. The complete list of selected genes with their annotated functions and primers are listed in Supplementary Table S1. A detailed explanation for the selection of these target genes are also provided for as Supplementary Information 1. *D. vulgaris* biofilms were propagated in anaerobic serum bottles (working volume of 120 mL) using both saline and freshwater media, with three biological replicates each. Submerged fed-batch biofilm reactors were used to propagate biofilms on cellulose acetate (CA) coupons (5 mm × 5 mm) fastened together on a sterile 4” 22G needle (Air-Tite, Virginia Beach, VA, United States) and each reactor had three of such networks (five coupons per network) of cellulose acetate coupons. Tests were conducted in three phases, with each phase lasting for 7 days. At the end of each phase, half of the spent medium was replaced with fresh medium. At the end of the final phase, planktonic cells and CA membrane coupon-bound biofilms in the reactor were harvested separately. Briefly, coupons from each reactor were placed in 10 mL 0.9% NaCl and then individually subjected to ultrasonication at 25% amplitude with 2 s pulsating intervals for 3 min using a water-bath sonicator (Q500, Qsonica, Newton, CT, United States) (Cheng et al., 2016). Dispersed biofilm cells as well as freely suspended planktonic cells in the reactor were harvested through centrifugation (8,000 × *g*, 10 min) and used for extracting RNA after treatment with RNA protect (Qiagen, Hilden, Germany). The cell-RNA protect mixture was incubated for 5 min at room temperature and then centrifuged at 8,000 × *g* for 15 min. RNA-protected treated cell pellets were then stored at −80°C until RNA extraction. RNA extraction was performed using RNeasy Mini kits (Qiagen, Hilden, Germany) according to the manufacturer’s protocol and RNA concentration was quantified using the Qubit 2.0 fluorometer (Thermo Fisher Scientific, San Jose, CA, United States) (Jumat et al., 2018). 1 μg of RNA extracts from biofilms were used as template for the synthesis of complementary DNA (cDNA) for RT-qPCR based on previously described protocols (Jumat et al., 2018). Target genes were amplified from the *D. vulgaris* genome using polymerase chain reaction (PCR). PCR products were cloned to pCR2.1 cloning vectors (Thermo Fisher Scientific, San Jose, CA, United States) and then transformed to *E. coli* TOP10 cells (Thermo Fisher Scientific, San Jose, CA, United States). The plasmids encoding each respective target gene were extracted using Plasmid Miniprep protocol (Promega, Madison, WI, United States). Based on the empirical relationship between plasmid DNA concentration, insert and vector size, the plasmid copy number was calculated. Plasmid DNA were then subjected to successive 10-fold serial dilutions to prepare the standard curve between the threshold cycle (*C*_T_) and plasmid DNA copy number. The amplification efficiency and regression coefficient (*R*^2^) corresponding to standard curves for each target gene are provided in Supplementary Table S1. The volumes of reagents used for RT-qPCR were as follows: Fast SYBR Green Master Mix (Thermo Fisher Scientific, San Jose, CA, United States), 10 μL; forward and reverse primers, 0.4 μL each; cDNA template, 1 μL and PCR grade H_2_O, 8.2 μL. RT-qPCR was conducted using Applied Biosystems^®^ QuantStudio 3 Real-Time PCR system (Thermo Fisher Scientific, San Jose, CA, United States). RT-qPCR cycle also included a melting curve analysis through an increase in temperature from 60 to 95°C for 5 s at 0.5°C interval. The copy numbers of each target gene estimated from the RT-qPCR standard curve were normalized against the single copy housekeeping gene Recombinase A *recA* (DVU1090). *recA* has displayed lower levels of gene expression heterogeneity in *D. vulgaris* biofilm growth mode, compared to other internal reference gene such as 16S rRNA (DV16SA) and glyceraldehyde 3-phosphate dehydrogenase (DVU0565), in accordance with previous studies (Zhang et al., 2007; Clark et al., 2012; Qi et al., 2014, 2016). Threshold cycle data values of *recA* extracted from *D. vulgaris* planktonic cells and biofilms are shown in Supplementary Table S2, and further demonstrated a low level of gene expression heterogeneity in this study.

### Quantification of Extracellular Polysaccharides and Proteins

Extracellular polysaccharides and proteins (EPS) from cellulose acetate membrane coupons-attached biofilms were detached through ultrasonication in 10 mL 0.9% NaCl, as mentioned in the previous section. After ultrasonication, the suspension harboring detached cells and EPS from CA membrane coupons was centrifuged (8,000 × *g*, 10 min). 0.25 μm syringe-filtered cell-free supernatant was used for quantification of EPS using liquid chromatography with organic carbon detector (LC-OCD) model-8 (DOC-Labor, Germany) equipped with a Toyopearl size exclusion chromatography column TSK HW50S (Tosoh, Japan) (Dimension: 250 mm × 20 mm, particle size: 20–40 μm). Polysaccharides and proteins from total EPS were fractionated and resolved using organic carbon detector (OCD) and organic nitrogen detector (OND). ChromCALC uni software was used to determine the concentration of each fraction in organic matter below the curve based on the integration of the defined area (Huber et al., 2011; Stewart et al., 2013).

### Evaluation of the Impacts of Quorum Sensing Inhibitors on SRB Planktonic Cells and Biofilms

To gain a better understanding of the role of QS in events leading to biocorrosion, three quorum sensing inhibitors (QSIs), (5Z)-4-bromo-5-(bromoethylene)-3-butyl-Z(5H)-furanone (bromofuranone), 3-oxo-D12-N-(2-oxocyclohexyl) dodecanamide (3-oxo-N) and γ-aminobutyric acid (GABA) (Sigma-Aldrich, MI, United States) were added to saline medium harboring *D. vulgaris* and *Db. corrodens* planktonic cells at different concentrations. Sub-inhibitory concentrations of QSI were selected from previous studies based on the effect of QSI on growth kinetics and biofilm formation (Hentzer et al., 2003; Smith et al., 2003; Ren and Wood, 2004; Chevrot et al., 2006). QSIs were deployed at sub-inhibitory concentrations (bromofuranone, 40 μM; 3-oxo-N, 20 μM; and GABA, 1 mM) and also at concentrations higher than sub-inhibitory concentrations, as follows: Bromofuranone (μM) – 80, 120, 160; 3-oxo-N (μM) – 40, 80, 120, 160; GABA (mM) – 1, 2, 5, 10, 20, 50. Planktonic SRB cells in saline medium without the addition of any QSI were used as a control in this study. Three biological replicates were maintained for each test condition, in a working volume of 22 mL. The effects of QSI on sulfate reduction, AHL production and specific growth rate were quantified. All concentrations of QSI described above were also used for biofilms to determine their effects on SRB biofilm formation.

### Statistical Analysis

All statistical assays were performed using Data Analysis tool on Microsoft Excel 2017. The degree of correlation in kinetics involving sulfate reduction, AHL production and cell density for all test conditions was measured in terms of Spearman’s rank correlation coefficient. The statistical significance tests were performed using two-tailed *t*-test on Microsoft Excel 2017.

## Results

### Salinity Enhances Biofilm Formation by *D. vulgaris* and *Db. corrodens* but Does Not Promote Growth

*D. vulgaris* exhibited similar specific growth rates under saline and freshwater conditions, respectively (saline, 0.17 ± 0.02/d; freshwater, 0.14 ± 0.02/d; *p* = 0.15) (Supplementary Figure S2A). Similar trend was also observed for *Db. corrodens* (saline, 0.17 ± 0.03/d; freshwater, 0.16 ± 0.02/d; *p* = 0.32) (Supplementary Figure S2B). However, salinity significantly improved biofilm formation by *D. vulgaris* and *Db. corrodens*. Under saline conditions, *D. vulgaris* produced 1.5-times higher biofilm biomass than freshwater conditions (saline, OD_590_ = 1.80 ± 0.48; freshwater, OD_590_ = 1.17 ± 0.38; *p* = 7.65 × 10^−6^). Similarly, salinity also enhanced biofilm biomass of *Db. corrodens* by1.6-times (saline, OD_590_ = 1.64 ± 0.43; freshwater, OD_590_ = 1.03 ± 0.18; *p* = 2.77 × 10^−6^). In addition, higher polysaccharide to protein ratio for both *D. vulgaris* (2.05-folds) (saline, 0.76 ± 0.11 μg/μg; freshwater, 0.37 ± 0.05 μg/μg; *p* = 0.02) and *Db. corrodens* (2.0-folds) (saline, 0.56 ± 0.01 μg/μg; freshwater, 0.28 ± 0.06 μg/μg, *p* = 0.03) biofilms was observed under saline conditions.

### Salinity Enhances Sulfate Reduction by *D. vulgaris* and *Db. corrodens*

**FIGURE 1:**
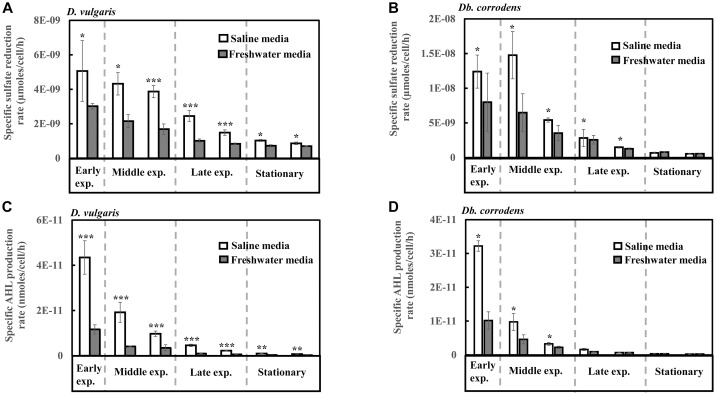
Specific sulfate reduction and specific AHL production rates displayed by *D. vulgaris* and *Db. corrodens* in saline and freshwater media. **(A)** Specific sulfate reduction rate exhibited by *D. vulgaris* planktonic cells. **(B)** Specific sulfate reduction rate exhibited by *Db. corrodens* planktonic cells. **(C)** Specific AHL production rate exhibited by *D. vulgaris* planktonic cells. **(D)** Specific AHL production rate exhibited by *Db. corrodens* planktonic cells. Results are presented as mean ± standard deviation (*n* = 3). Significant difference: ^∗^*p* < 0.05; ^∗∗^*p* < 0.01; ^∗∗∗^*p* < 0.001. Early exp. corresponds to early exponential phase; Middle exp. corresponds to middle exponential phase; and Late exp. corresponds to late exponential phase.

Under saline conditions, *D. vulgaris* displayed significantly higher specific sulfate reduction rate (ca. 1.4- to 2.5-times, *p* < 0.05) during early (24 h), middle (48–72 h) and late exponential phases (96–120 h) as well as stationary phase (144–168 h) (Figure 1A) compared to that observed under freshwater conditions. Similarly, high specific sulfate reduction rates (ca. 1.3- to 2.3-times, *p* < 0.05) were observed for *Db. corrodens* under saline conditions during exponential phases (Figure 1B). However, *Db. corrodens* exhibited similar specific sulfate reduction rates during stationary phase (144–168 h, *p* > 0.05) under saline and freshwater conditions (Figure 1B).

### Salinity Increases AHL Production by *D. vulgaris* and *Db. corrodens*

In saline medium, total AHLs for *D. vulgaris* ranged from 20 to 27 nM (Supplementary Figure S2C) and 6 to 10 nM for *Db. corrodens* during exponential and stationary phases. This amount is higher, when compared to freshwater conditions (*D. vulgaris*, 12–14 nM; *Db. corrodens*, 4–6 nM) (Supplementary Figures S2C,D). Under saline conditions, *D. vulgaris* exhibited ca. three to four times higher specific AHL production rate for all growth phases as compared to freshwater conditions (*p* < 0.05, Figure 1C). In the case of *Db. corrodens*, an increase in specific AHL production rate by ca. 1.5- to 2-times was observed between early to mid-exponential phases in saline medium (*p* < 0.05) but the significant difference was no longer apparent in the latter growth phases (Figure 1D).

### High Correlation Between Sulfate Reduction and AHL Production Under Saline Conditions

A higher correlation between specific sulfate reduction rate and specific AHL production rate was observed for *D. vulgaris* (*R*^2^ = 0.87; *p* = 0.01) under saline conditions compared to freshwater conditions (*R*^2^ = 0.75; *p* = 0.01). Similarly, *Db. corrodens* exhibited higher correlation between specific sulfate reduction rate and specific AHL production rate in saline medium (*R*^2^ = 0.93; *p* = 0.03) compared to freshwater medium (*R*^2^ = 0.73; *p* = 0.01). In addition, both *D. vulgaris* and *Db. corrodens* displayed a higher correlation between sulfate reduction and AHL production during early and mid-exponential phase (*R*^2^≥ 0.79; *p* < 0.05) compared to late exponential and stationary phases (*R*^2^≤ 0.63; *p* < 0.05) in saline medium.

### RT-qPCR Analysis Reveals an Increase in the Expression Levels of Targeted Genes Under Saline Conditions

**FIGURE 2:**
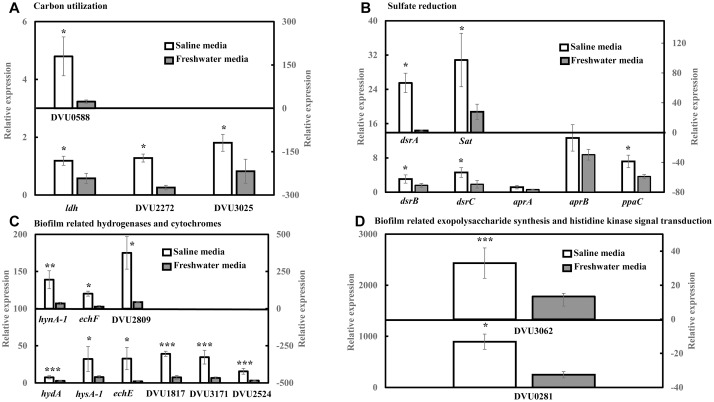
RT-qPCR analysis of relative expression of selected genes related to carbon metabolism, sulfate reduction, electron transfer and biofilm formation in *D. vulgaris* biofilms under saline and freshwater conditions**. (A)** Relative expression of carbon metabolism enzymes lactate dehydrogenase *ldh*, pyruvate formate lyase DVU2272 and pyruvate dehydrogenase DVU3025 in the primary left *y*-axis, and formate dehydrogenase DUV0588 in the secondary right *y*-axis. **(B)** Relative expression of dissimilatory sulfite reductase *dsrB*, *dsrC*, adenosine 5′-phosphosulfate reductase *aprA*, *aprB* and pyrophosphatase *ppaC* in the primary left *y*-axis, and dissimilatory sulfite reductase alpha subunit *dsrA* and sulfate adenylytransferase *Sat* in the secondary right *y*-axis. **(C)** Relative expression of Fe hydrogenase *hydA*, NiFeSe hydrogenase *hysA*-1, Ech hydrogenases *echE*, formate dehydrogenase DVU1817and *c*3-type cytochromes DVU3171 and DVU2524 in the primary left *y*-axis, as well as NiFe hydrogenase *hynA*-1, Ech hydrogenase *echF*, and *c*3-type cytochrome DVU2809 in the secondary right *y*-axis. **(D)** Relative expression of exopolysaccharide synthesis protein DVU0281 in the primary left *y*-axis and sensor histidine kinase response regulator DVU3062 in the secondary right *y*-axis. Relative expression refers to the transcript level of a specific gene normalized with that of reference gene *recA*. Results are presented as mean ± standard deviation (*n* = 3). Significant difference: ^∗^*p* < 0.05; ^∗∗^*p* < 0.01; ^∗∗∗^*p* < 0.001.

RT-qPCR was conducted to quantify the abundance of specific genes related to sulfate reduction, carbon metabolism, hydrogenases and cytochromes, exopolysaccharide synthesis and signal response regulator in *D. vulgaris* biofilms propagated under saline and freshwater conditions. Key functions of all the respective genes are listed in Supplementary Table S1. Compared to freshwater conditions, expression levels of genes related to lactate metabolism like lactate dehydrogenase *ldh* (2.04-folds; *p* = 0.03) and pyruvate: ferredoxin oxidoreductase DVU3025 (2.19-folds; *p* = 0.04) were significantly upregulated under saline conditions (Figure 2A). High relative expression of genes involved in pyruvate and formate cycling such as pyruvate formate lyase DVU2272 (4.96-folds; *p* = 0.02) and formate dehydrogenases DVU0588 (7.71-folds; *p* = 0.02) was also detected under saline conditions (Figure 2A). The expression of all dissimilatory sulfite reductase subunits such as dissimilatory sulfite reductase alpha subunit *dsrA* (25-folds; *p* = 0.02), *dsrB* (1.92-folds; *p* = 0.05) and *dsrC* (2.49-folds; *p* = 0.05) was upregulated in saline conditions in *D. vulgaris* biofilms (Figure 2B). Although, no significant induction was detected for adenosine 5′-phosphosulfate reductase *aprA* and *aprB* (*p* > 0.05) (Figure 2B), sulfate adenyltransferase Sat, a key player in sulfate reduction was significantly upregulated (3.50-folds; *p* = 0.03) under saline conditions (Figure 2B). High abundance of periplasmic Fe hydrogenase alpha subunit *hydA* (2.73-folds; *p* = 7.40 × 10^−5^), NiFe hydrogenase alpha subunit *hynA*-1 (5.61-folds; *p* = 2.20 × 10^−3^) and NiFeSe hydrogenase *hysA*-1 (4.12-folds; *p* = 0.02) as well as Ech hydrogenases *echE* (14.88-folds; *p* = 0.03), echF (7.45-folds; *p* = 0.03) and cytochrome *c*553 DVU1817 (5.22-folds; *p* = 6.30 × 10^−3^) was also observed under saline conditions (Figure 2C). In addition, *c*3-type cytochromes harboring heme groups such as DVU3171 (5.14-folds; *p* = 7.07 × 10^−3^), DVU2524 (4.92-folds; *p* = 8.03 × 10^−3^) and DVU2809 (8.57-folds; *p* = 0.04) were also found to be significantly upregulated in the presence of salinity (Figure 2C). Lastly, salinity enhanced the expression of DVU0281 (3.58-folds; *p* = 0.04), which encodes for exopolysaccharide synthesis and DVU3062 (2.47-folds; *p* = 9.60 × 10^−3^), a histidine kinase involved in intracellular communication (Figure 2D). Apparently, expression of target genes was also found to be upregulated in case of *D. vulgaris* planktonic cells extracted from the biofilm reactor (Supplementary Figure S3).

### Quorum Sensing Inhibitors Decrease Specific Growth Rates and Biofilm Formation of SRB in Saline Media

**FIGURE 3:**
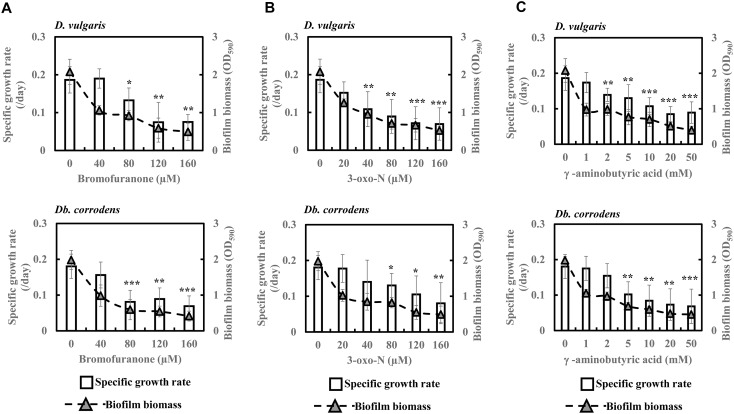
Quorum sensing inhibitors (QSIs) and the effect on specific growth rate and biofilm formation of *D. vulgaris* and *Db. corrodens* in saline media. **(A)** Effect of bromofuranone on specific growth rate and biofilm formation of *D. vulgaris* (upper panel) and *Db. corrodens* (lower panel). **(B)** Effect of 3-oxo-N on specific growth rate and biofilm formation of *D. vulgaris* (upper panel) and *Db. corrodens* (lower panel). **(C)** Effect of γ-aminobutyric acid (GABA) on specific growth rate and biofilm formation of *D. vulgaris* (upper panel) and *Db. corrodens* (lower panel). Bar chart illustrates specific growth rate plot and dotted line scatter plot illustrates biofilm biomass plot. Results are presented as mean ± standard deviation (*n* = 3). Significant difference in specific growth rate: ^∗^*p* < 0.05; ^∗∗^*p* < 0.01; ^∗∗∗^*p* < 0.001.

To further comprehend and establish the linkage between QS and sulfate reduction in SRB, *D. vulgaris* and *Db. corrodens* were propagated in saline media in the presence and absence of QSIs. Figure 3 shows specific growth rate and biofilm biomass of *D. vulgaris* and *Db. corrodens* in saline medium in the presence and absence of QSI. Specific growth rate of *D. vulgaris* decreased significantly (ca. 1.41 to 2.65-times; *p* < 0.05) in the presence of bromofuranone ≥ 80 μM compared to the control (Figure 3A). The addition of 3-oxo-N ≥ 40 μM (ca. 1.72- to 2.71-times; *p* < 0.05) (Figure 3B) and GABA ≥ 5 mM (ca. 1.43- to 2.08-times; *p* < 0.05) (Figure 3C) resulted in similar decrease of specific growth rates of *D. vulgaris*. Likewise, at similar inhibitory concentrations, bromofuranone (ca. 2.35-times; *p* < 0.05), 3-oxo-N (ca. 1.33 to 2.32-times; *p* < 0.05) and GABA (ca. 1.84 to 2.70-times; *p* < 0.05) significantly decreased the specific growth rate of *Db. corrodens* (Figure 3C). Biofilm formation by *D. vulgaris* and *Db. corrodens* was compromised (*p* < 0.05) even at bromofuranone ≤ 40 μM, 3-oxo-N ≤ 20 μM and GABA ≤ 2 mM, as illustrated by the sharp decrease in biofilm biomass compared to control (Figure 3).

### Quorum Sensing Inhibitors Inhibit Sulfate Reduction by SRBs in Saline Media

**FIGURE 4:**
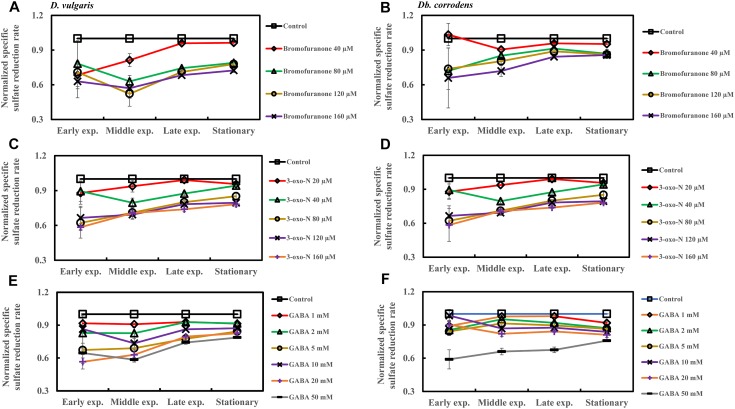
Quorum sensing inhibitors (QSIs) and the effect on sulfate reduction by *D. vulgaris* and *Db. corrodens* in saline media. Specific sulfate reduction rate of QSI-treated *D. vulgaris* and *Db. corrodens* in saline medium was normalized with that of control (no QSI added) and plotted along *y*-axis. **(A)** Effect of bromofuranone on specific sulfate reduction rate exhibited by *D. vulgaris* planktonic cells in saline medium. **(B)** Effect of bromofuranone on specific sulfate reduction rate exhibited by *Db. corrodens* planktonic cells in saline medium. **(C)** Effect of 3-oxo-N on specific sulfate reduction rate exhibited by *D. vulgaris* planktonic cells in saline medium. **(D)** Effect of 3-oxo-N on specific sulfate reduction rate exhibited by *Db. corrodens* planktonic cells in saline medium. **(E)** Effect of γ-aminobutyric acid (GABA) on specific sulfate reduction rate exhibited by *D. vulgaris* planktonic cells in saline medium. **(F)** Effect of GABA on specific sulfate reduction rate exhibited by *Db. corrodens* planktonic cells in saline medium. Results are presented as mean ± standard deviation (*n* = 3). Early exp. corresponds to early exponential phase; Middle exp. corresponds to middle exponential phase; Late exp. corresponds to late exponential phase.

The effect of QSI with increasing concentrations on specific sulfate reduction rate during early, middle, late exponential and stationary phases is shown in Figure 4. Addition of bromofuranone ≥ 80 μM significantly decreased the specific sulfate reduction rate of *D. vulgaris* to ca. 0.52- to 0.71-times that of control (*p* < 0.05) (Figure 4A and Supplementary Table S3), while specific reduction rate of *Db. corrodens* decreased to ca. 0.72- to 0.84-times that of control (*p* < 0.05) during exponential phase (Figure 4B and Supplementary Table S3). In the presence of 3-oxo-N ≥ 40 μM, the specific sulfate reduction rate of *D. vulgaris* dropped to ca. 0.62 to 0.78-times of control (*p* < 0.05) (Figure 4C and Supplementary Table S3). The same is observed for *Db. corrodens* in the presence of 3-oxo-N during exponential phase (Figure 4D and Supplementary Table S3). Similarly, the specific sulfate reduction rate of *D. vulgaris* was ca. 0.58 to 0.83-times of control (*p* < 0.05) (Figure 4E and Supplementary Table S3) and that of *Db. corrodens* was ca. 0.60 to 0.84-times of control (*p* < 0.05) (Figure 4F and Supplementary Table S3) when exposed to GABA ≥ 5 mM. During stationary phase, decrease in specific sulfate reduction rate displayed by QSI-treated *D. vulgaris* and *Db. corrodens* was marginal (ca. 0.75- to 0.95-times of control; *p* > 0.05) compared to exponential phase (Figure 4 and Supplementary Table S3).

### Quorum Sensing Inhibitors Inhibit AHL Production by SRBs in Saline Media

**FIGURE 5:**
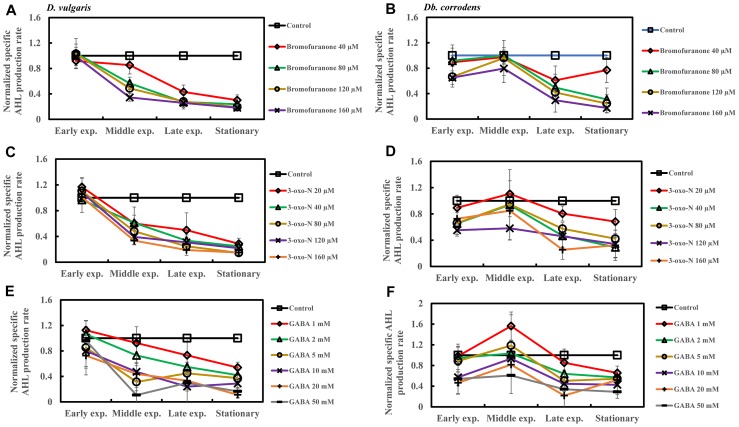
Quorum sensing inhibitors (QSIs) and the effect on AHL production by *D. vulgaris* and *Db. corrodens* in saline media. Specific AHL production rate of QSI-treated *D. vulgaris* and *Db. corrodens* in saline medium was normalized with that of control (no QSI added) and plotted along *y*-axis. **(A)** Effect of bromofuranone on specific AHL production rate exhibited by *D. vulgaris* planktonic cells in saline medium. **(B)** Effect of bromofuranone on specific AHL production rate by *Db. corrodens* planktonic cells in saline medium. **(C)** Effect of 3-oxo-N on specific AHL production rate exhibited by *D. vulgaris* planktonic cells in saline medium. **(D)** Effect of 3-oxo-N on specific AHL production rate exhibited by *Db. corrodens* planktonic cells in saline medium. **(E)** Effect of γ-aminobutyric acid (GABA) on specific AHL production rate exhibited by *D. vulgaris* planktonic cells in saline medium. **(F)** Effect of GABA on specific AHL production rate exhibited by *Db. corrodens* planktonic cells in saline medium. Results are presented as mean ± standard deviation (*n* = 3). Early exp. corresponds to early exponential phase; Middle exp. corresponds to middle exponential phase; Late exp. corresponds to late exponential phase.

Addition of bromofuranone (≥80 μM), 3-oxo-N (≥40 μM), and GABA (≥5 mM) to *D. vulgaris* reduced the specific AHL production rate to ca. 0.2- to 0.40-times of control during middle and late-exponential phases (*p* < 0.05) and to ca. <0.25-times of control during stationary phase (*p* < 0.05) (Figure 5A,C,E and Supplementary Table S4). Similarly, bromofuranone (≥80 μM), 3-oxo-N (≥40 μM) and GABA (>5 mM) considerably decreased the specific AHL production rate of *Db. corrodens* during middle and late exponential phases (ca. <0.50-times of control; *p* < 0.05) and stationary phase (ca. <0.40-times of control; *p* < 0.05) (Figure 5B,D,F and Supplementary Table S4).

Increasing concentrations of QSIs (bromofuranone ≥ 80 μM; 3-oxo-N ≥ 40 μM and GABA ≥ 5 mM) decreased the overall correlation (*R*^2^) between specific sulfate reduction rate and specific AHL production rate from 0.87 to 0.57–0.78 for *D. vulgaris* (*p* < 0.05) and to 0.54–0.73 for *Db. corrodens* (*p* < 0.05). Likewise, QSIs also decreased the correlation between specific sulfate reduction rate and specific AHL production rate during early and mid-exponential phases from 0.79 to a range of 0.27–0.56. This decrease in correlation was more apparent in the exponential phases compared to that observed during stationary phase.

## Discussion

Salinity is a key factor regulating the corrosion potential of a particular matrix. Increasing levels of salinity shifts corrosion potential in negative direction and hence, is often accompanied with increase in corrosion rates (Mansfeld et al., 2002). At the same time, saline environment favors the proliferation of SRBs such as *D. vulgaris* and *Db. corrodens* because of their ability to tolerate high salt stress (Lovley and Phillips, 1994; Blessing et al., 2001; Mukhopadhyay et al., 2006). An earlier study reported an increase in SRB cell numbers when salinity was increased from 13 g/L to 35 g/L, and a decline in SRB numbers as salinity increased further from 35 g/L to 80 g/L (hypersaline range). Coincidentally, biocorrosion rate was also highest when salinity was 35 g/L and when SRB were most abundant (De França et al., 2000). However, the earlier study only reported the overall sulfate reduction rates and did not normalize against cell numbers to obtain the specific sulfate reduction rates that would be more indicative of the sulfate reduction activity per cell.

In this study, it was first observed that the biofilm formation by *D. vulgaris* and *Db. corrodens* was higher in saline media than in freshwater media even though the specific growth rates of both SRB in both media were similar. It was then observed that the specific sulfate reduction rates were also higher in the saline media than in the freshwater media (Figure 1A,B), and that the higher specific sulfate reduction rate was accounted for by a higher expression of sulfate reduction genes in the saline media than in the freshwater media (Figure 2).

Although the mechanisms triggering the increased expression of sulfate reduction genes under saline conditions are not known, we infer that certain genes with possible dual roles in salinity tolerance and sulfate reduction were triggered by salinity. For example, oxidoreductases are often reported to be regulated by increasing saline content in media since oxidoreductases either serve as sensors or contribute to bacterial tolerance under saline environments (Bhatt and Weingart, 2008; Pumirat et al., 2010). Based on previous studies, the expression levels of NADH-dependent oxidoreductases such as lactate dehydrogenase, formate dehydrogenase, and succinate dehydrogenase were upregulated under saline conditions (Fu et al., 1989; Weerakoon et al., 2009; Pumirat et al., 2014). This corroborates with the finding of this study related to the upregulation of lactate dehydrogenase *ldh*, formate dehydrogenase DVU0588 and DVU1817 and pyruvate dehydrogenase DVU3025 by *D. vulgaris* biofilm cells under saline conditions. The increased expression of *ldh* and DVU3025 under saline conditions can subsequently lead to improved electron flow and overall metabolic activity (Heidelberg et al., 2004; Keller and Wall, 2011). Furthermore, sulfate reductive enzymes have been reported to be highly dependent on carbon metabolism genes (Pereira et al., 2008; Keller and Wall, 2011). This might explain the increase in specific sulfate reduction rates of *D. vulgaris* in saline media compared to freshwater media.

In addition, the upregulation of Ech hydrogenases as well as *c*3-type cytochromes likely suggest an improved electron flow within *D. vulgaris* under saline environment. The induction of formate dehydrogenases and Ech hydrogenases under saline conditions is consistent with that reported by earlier studies (Mukhopadhyay et al., 2006; Clark et al., 2012). It is therefore inferred that salinity elevates the expression of carbon metabolism enzymes and electron transfer machinery within SRB, which in turn leads to enhanced specific sulfate reduction rates as observed in this study and indirectly accelerating rates of SRB-mediated biocorrosion in seawater environments.

Coincidentally, the increase in both biofilm formation and specific sulfate reduction rates in both SRB species were observed along with an increase in the specific AHL production rates (Figure 1C,D), suggesting a potential connection between AHL and sulfate reduction. This is especially so during the early and mid-exponential phases, likely when carbon metabolism of SRBs is most active. Previous studies have demonstrated the production of AHLs by SRB (Decho et al., 2009) but their exact role within SRB was not elucidated. Signal molecules extracted from SRB within microbial mats have been implicated to be the driving force for metabolic activities and interspecies interactions within microbial mats (Decho et al., 2009, 2010) but no prior studies have demonstrated the inter-connection between AHL production, biofilm formation and sulfate reduction.

This study has demonstrated a potential link between AHL production, biofilm formation and sulfate reduction among SRBs under saline conditions. To an extent, enhanced expression of biofilm related genes and sulfate reductive enzymes under saline conditions allude toward interconnection between QS and sulfate reduction at transcriptomic level. However, the choice of biofilm related genes considered for this study might be limited to underpin the nature of this interconnection at molecular level. Based on our findings, it could be speculated that addition of AHLs extracted from SRBs might improve the overall specific sulfate reduction rate by *D. vulgaris* and *Db. corrodens.* Further studies that monitor the effects of exogenously added AHLs on sulfate reduction, possibly using transcriptomics approaches, could provide a more comprehensive means to establish the nature of interconnection between QS and sulfate reduction among SRBs.

Nevertheless, this study attempts to further verify the connection between AHL and sulfate reduction by applying QSI at varying concentrations. A decrease in specific sulfate reduction rates was observed during the exponential phase of SRB (Figure 4 and Supplementary Table S3). This reduction in specific sulfate reduction rate was also accompanied by a considerable decline in both specific AHL production rate (Figure 5 and Supplementary Table S4) and biofilm formation (Figure 3). The effect imposed by QSI was however not apparent during the late exponential or stationary phase. The findings collectively suggest that QS pathway could contribute in enhancing specific sulfate reduction rates of metabolically active SRB that propagate in saline environment. However, the exact pathways modulated by the QS mechanisms remain unknown.

Previous studies have reported the use of natural or synthetic compounds in saline conditions to quench QS in bacteria such as *Vibrio* (*V.*) *harveyi*, *V. vulnificus*, *Halomonas pacific* and complex microbial community attached to reverse osmosis membrane (Shen et al., 2006; Liaqat et al., 2014; Mai et al., 2015; Santhakumari et al., 2016). In those instances, the use of QSI demonstrated strong inhibition on biofilm formation. However, those studies did not evaluate if QSI approaches would be suitable to inhibit SRBs, and the associated sulfate reduction and biocorrosion rates. This is likely due to the lack of understanding on whether QS is indeed present among SRBs (Scarascia et al., 2016) and if present, whether there is a correlation with specific sulfate reduction rates. This study demonstrated that SRB biofilms are highly susceptible to QSI application, and a consequential decrease in specific sulfate reduction rates can indeed be achieved. Hence, QSI could be deployed as potential biocides to inhibit SRB biofilm-mediated biocorrosion during the early phases of biofilm formation. The efficacy of QSI however may be low on mature SRBs and biofilm.

## Conclusion

In summary, by using *D. vulgaris* and *Db. corrodens* as model SRBs, we showed that saline conditions significantly increase the rates of specific sulfate reduction, AHL production and biofilm formation by *D. vulgaris* and *Db. corrodens*. By deploying QSIs, a potential connection between sulfate reduction and AHL production under saline conditions was demonstrated, which is most significant during early stages of sulfate metabolism. Insights from this study revealed the interconnection between QS, sulfate reduction and biofilm formation among SRBs. Furthermore, this study showed quorum quenching molecules could be deployed as an environmentally benign approach to control SRB at the early stages of growth and biofilm formation.

## Author Contributions

KS designed and performed the experiments, data analysis and wrote the manuscript. GS contributed to extraction and analysis of total AHLs and sulfate reduction. TW developed the protocol for quantification and analysis of total AHLs using bioluminescence assay and LC-MS. NZ conducted quantification of extracellular polysaccharides and proteins. AK provided advice for cultivating sulfate reducing bacteria and comments on the manuscript. P-YH conceived and designed the experiments, analysis and interpretation of data, wrote the manuscript, supervised the research, and provided reagents and materials.

## Conflict of Interest Statement

The authors declare that the research was conducted in the absence of any commercial or financial relationships that could be construed as a potential conflict of interest.
