# Supplementary material for: Salinity-Mediated Increment in Sulfate Reduction, Biofilm Formation, and Quorum Sensing: A Potential Connection Between Quorum Sensing and Sulfate Reduction?
Source: Front Microbiol. 2019 Feb 6;10:188. doi: 10.3389/fmicb.2019.00188 (PMC6373464; doi:10.3389/fmicb.2019.00188)
Supplement: Supplementary file 1 [file Data_Sheet_1.docx]

**Salinity-mediated increment in sulfate reduction, biofilm formation and quorum sensing: a potential connection between quorum sensing and sulfate reduction?**

***Krishnakumar Sivakumar^1^, Giantommaso Scarascia^1^, Noor Zaouri^1^, Tiannyu Wang^1^,***

***Anna H. Kaksonen^2^, Pei-Ying Hong*^1^***

*^1^Water Desalination and Reuse Center (WDRC), Biological and Environmental Sciences and Engineering Division (BESE), King Abdullah University of Science and Technology (KAUST), Thuwal-Jeddah 23955-6900, Saudi Arabia*

*^2^ Commonwealth Scientific and Industrial Research Organization (CSIRO) Land and Water, Floreat, Western Australia 6014, Australia*

*Tel: +966-(12)-808-2218; Email: [peiying.hong@kaust.edu.sa](mailto:peiying.hong@kaust.edu.sa)

**Supplementary Information 1. Selection of target genes for RT-qPCR analysis**

To understand the molecular mechanism underlining salinity-enhanced QS and biofilm formation, we adopted RT-qPCR approach to profile the expression levels of certain specific genes that displayed higher gene expression heterogeneity under biofilm growth mode. Genes targeted for RT-qPCR analysis, listed in Supplemental Table S1 were made from previous studies. Dissimilatory sulfate reduction by *D. vulgaris* is a 3-step reaction mediated by soluble reductive enzymes. In the first step, sulfate adenylyltransferase (Sat) converts sulfate to adenosine 5’-phosphosulfate (APS) with pyrophosphate as byproduct. Inorganic pyrophosphatase (PpaC) hydrolyzes pyrophosphate releasing inorganic phosphates as a secondary reaction. APS is reduced to sulfite by APS reductases (AprAB) and dissimilatory sulfite reductases (DsrABC) mediates the reduction of sulfite to sulfide (Keller and Wall, 2011a). Lactate dehydrogenase (Ldh) and pyruvate: ferredoxin oxidoreductase (DVU3025) play a key role in oxidation of lactate (McInerney and Bryant, 1981;Keller and Wall, 2011a). Pyruvate and formate cycling mediated by enzymes such as pyruvate formate lyase (DVU2272) and formate dehydrogenases (DVU0588) has been reported to be a key mechanism in electron flow within lactate-respiring *D. vulgaris* planktonic cells and biofilms (Zhang et al., 2007;Clark et al., 2012). SRBs harbor three classes of periplasmic hydrogenases grouped as [Fe], [NiFe] and [NiFeSe] encoded by operons containing *hydAB*, *hynBA* and *hysBA* respectively and are supposed to be the driving force in anaerobic biocorrosion mediated by SRBs (Voordouw et al., 1990;Keller and Wall, 2011a). Ech hydrogenases facilitates the reduction of ferredoxin with hydrogen and in turn contributes to intracellular electron transfer. Ech hydrogenases, c-type cytochromes and cytochrome c553 play a crucial role in biofilm formation and attachment, metal reduction and extracellular electron transfer in SRB (Clark et al., 2012;Scarascia et al., 2016).

Extensive genomic mining has revealed 64 putative sensor histidine kinases and 72 response regulators, which comprises the overall two-component signal transduction systems within *D. vulgaris* genome (Zhang et al., 2006;Zhang et al., 2007;Rajeev et al., 2011). However, none of these have shown homology with known biofilm-induced regulators such as RpoS and CpsR (Zhang et al., 2007). Although gene expression levels of more than dozen signal genes varied significantly between *D. vulgaris* biofilms and planktonic cells, previous studies have underpinned a hybrid-type kinase DVU3062, which harbors histidine and aspartate domains to be the crucial signal transduction system involved in cell-cell communication within *D. vulgaris* (Zhang et al., 2006;Zhang et al., 2007;Qi et al., 2016). Hybrid-type kinases have been reported to be involved in cell-cell communication in other bacteria (Slater et al., 2000;Takeda et al., 2001;Zhang et al., 2006;Zhang et al., 2007). Given the lack of adequate information on QS in SRB, hybrid-type kinase signal transduction system DVU3062 was selected. Further, *D. vulgaris* biofilms have been reported to display upregulated levels of DVU0281, which encodes for exopolysaccharide synthesis. It also has been proposed that DVU0281 and DVU3062 might be involved in the formation and metabolism of *D. vulgaris* biofilms (Zhang et al., 2007;Qi et al., 2014).

**Supplemental Table S1**: List of *D. vulgaris* genes selected for RT-qPCR study with respective primers, amplification efficiency and regression coefficient of standard curves

| **Locus Tag DVU No** | **Gene** | **Annotation** | | **Forward primer** | **Reverse primer** | **RT-qPCR amplification efficiency** | **RT-qPCR-based standard curve regression coefficient (R^2^)** | **Function** |
| --- | --- | --- | --- | --- | --- | --- | --- | --- |
| **Sulfate reduction enzymes** | | | | | | | | |
| 0402 | dsrA | Dissimilatory sulfite reductase alpha subunit | | GTGACCAGCCCGAAAAGTTC | CAAGGTCGGTGTTCAGGTTG | 107.78 | 0.9984 | Mediates the reduction of sulfite to sulfide in sulfate reduction pathway (Keller and Wall, 2011a;Clark et al., 2012) |
| 0403 | dsrB | Dissimilatory sulfite reductase beta subunit | | ATGTCGATCACCCACATCCG | ATGGGGAACTTGTAGGAGCC | 105.29 | 0.9969 |  |
| 2776 | dsrC | Dissimilatory sulfite reductase gamma subunit | | GCTGAAGTCACTTACAAGGGC | CCGGTGTTCTTCGAGAGGAT | 100.15 | 0.9889 |  |
| 0847 | aprA | Adenylsulfate reductase alpha subunit | | TAACCACATGATGCTTCGCG | ATGATTTCAGAACCGCGCTC | 101.75 | 0.9905 | Mediates the reduction of adenosine 5’-phosphosulfate to sulfite (Keller and Wall, 2011a;Clark et al., 2012) |
| 0846 | aprB | Adenylsulfate reductase beta subunit | | CATGTACATCTGCCCCAACG | TGGGGAACTTGAAACGCTTG | 100.02 | 0.9898 |  |
| 1295 | Sat | Sulfate adenylyltransferase | | CCTCCAGATGCGTGATGTTG | TCACTTTGATCCCGGACGG | 97.27 | 0.9953 | Reduction of sulfate to APS (Keller and Wall, 2011a) |
| 1636 | ppaC | Pyrophosphatase | | GTGAAGTCCGCTGTTGAAGG | GCATTTCCGAGCCTTCCTTC | 104.74 | 0.9936 | Hydrolysis of pyrophosphate (Keller and Wall, 2011a) |
| **Carbon utilization enzymes** | | | | | | | | |
| 0600 | ldh | Lactate dehydrogenase | | TATGAGCAGTGCGAAGGGG | ACATGGGTGAGGACATCGAC | 97.93 | 0.9951 | Lactate oxidation (McInerney and Bryant, 1981;Keller and Wall, 2011a;Meyer et al., 2014) |
| 2272 | DVU 2272 | Pyruvate formate lyase | | TGGATCAAGCACCTCGAACT | TACGAGTCCTTCAGCTTCGG | 105.03 | 0.9974 | Pyruvate cycling (Zhang et al., 2007;Pereira et al., 2008) |
| 3025 | DVU3025 | Pyruvate ferredoxin oxidoreductase | | CTGGTGAACTGGGAGAAGGT | GAAGGGCTTGTACTTGCGAC | 102.41 | 0.9942 | Lactate oxidation (McInerney and Bryant, 1981;Keller and Wall, 2011a;Meyer et al., 2014) |
| 0588 | DUV0588 | Formate dehydrogenase beta subunit | | GCATCGACTGGCTGTTCTTC | ACCCTGTCATTGCACATGTC | 99.57 | 0.9963 | Formate cycling (Zhang et al., 2007;Clark et al., 2012) |
| **SRB biofilm-related hydrogenases and cytochromes** | | | | | | | | |
| 1769 | hydA | | Periplasmic Fe hydrogenase alpha subunit | CGAGACCTACTACCCCGAAC | TGATCATGTAGGCCAGCTCA | 97.78 | 0.9922 | Anaerobic metabolism and intracellular electron transfer (Voordouw et al., 1990;Keller and Wall, 2011b) |
| 1922 | hynA-1 | | Periplasmic NiFe hydrogenase alpha subunit | GTCACCCCGCCTACTATCTC | GTCGGTGAATTCCTGGATGC | 104.65 | 0.9904 |  |
| 1918 | hysA-1 | | Periplasmic NiFeSe hydrogenase alpha subunit | ACCAGTACATCGAAGCCCTC | TACTTGCTGCCGATGGTGTA | 100.89 | 0.9878 |  |
| 0430 | echE | | Ech hydrogenase putative subunit EchE | ATCCGCGACTACAACCAGAT | CGCACTTTCCAGAACTGCAT | 98.56 | 0.9954 | Intracellular electron transfer (Clark et al., 2012) |
| 0429 | echF | | Ech hydrogenase putative subunit EchF | GCAACGTCCTCAAGAACCTG | TACAGAGCAGTAGACGCAGG | 101.42 | 0.9958 |  |
| 1817 | DVU1817 | | Cytochrome *c*553 putative formate dehydrogenase | ACGAGTTCTGCTTTTGTCCAG | TCTTGTACAGTTCCTCGGCC | 95.39 | 0.9972 | Biofilm formation and metal reduction (Zhang et al., 2007;Clark et al., 2012) |
| 3171 | DVU3171 | | *c*3- cytochrome (four hemes) | GCAAGGAAGACTACCGCAAG | CGTGGCACTTGGACTTCTTG | 97.94 | 0.9920 | Extracellular electron transfer and biofilm formation (Heidelberg et al., 2004;Keller and Wall, 2011a;Keller and Wall, 2011b) |
| 2524 | DVU2524 | | NiFe hydrogenase-associated *c*3- cytochrome (four hemes) | GCTGTTGTCTCCTGCACTAG | AGAAGCTCATCTGTTCGGCA | 98.78 | 0.9988 |  |
| 2809 | DVU2809 | | Formate dehydrogenase-associated *c*3- cytochrome (four hemes) | CGGATGCACGTGATGTTCAA | CATCTTCTTGTGGCAACCGT | 105.98 | 0.9982 |  |
| **SRB biofilm-related exopolysaccharide synthesis and sensor histidine kinase response regulator** | | | | | | | | |
| 0281 | DVU0281 | Exopolysaccharide biosynthesis protein | | TGTACTCTCGCCTGACCTTC | CCGAAATCATGGCATGTGGA | 103.52 | 0.9862 | Polysaccharide synthesis (Clark et al., 2007;Zhang et al., 2007;Clark et al., 2012;Qi et al., 2016) |
| 3062 | DVU3062 | Sensor histidine kinase response regulator | | TTCTGGACATTTCACTGCGC | AGCACATCCTCCACAGACTC | 97.89 | 0.9892 | Cell-cell communication (Zhang et al., 2007;Clark et al., 2012;Qi et al., 2016) |
| Reference gene | | | | | | | | |
| 1090 | recA | Recombinase A | | GCCCTGTTCGACATCCTCTA | GGAGTCATGCCAAGGTGTTC | 99.44 | 0.9970 | Reference gene (Zhang et al., 2007;Clark et al., 2012;Qi et al., 2016) |

**Supplemental Table S2**. Threshold cycle (C_t_) values of housekeeping gene recombinase A *recA* (DVU1090) exhibited by *D. vulgaris* biofilms and planktonic cells.

| **Growth mode** | **Saline media** | | | | | **Freshwater media** | | | | |
| --- | --- | --- | --- | --- | --- | --- | --- | --- | --- | --- |
|  | **C_t_-1** | **C_t_-2** | **C_t_-3** | **Mean C_t_** | **Standard deviation** | **C_t_-1** | **C_t_-2** | **C_t_-3** | **Mean C_t_** | **Standard deviation** |
| Biofilms | 20.58 | 20.67 | 20.81 | 20.68 | 0.09 | 20.44 | 20.45 | 21.08 | 20.66 | 0.30 |
| Planktonic cells | 21.02 | 20.84 | 20.74 | 20.86 | 0.12 | 20.69 | 20.74 | 20.44 | 20.63 | 0.13 |

**Supplemental Table S3.** Specific sulfate reduction rate exhibited by QSI-treated *D. vulgaris* and *Db. corrodens* planktonic cells in saline medium during early, mid, late exponential phases and stationary phase. No QSI was added to control.

| QSI concentra-tion | Specific sulfate reduction rate exhibited by QSI-treated *D. vulgaris* (×10^-12^ mmoles/cells/h) | | | | Specific sulfate reduction rate exhibited by QSI-treated *Db. corrodens* (×10^-12^ mmoles/cells/h) | | | | | |
| --- | --- | --- | --- | --- | --- | --- | --- | --- | --- | --- |
|  | **Early exp. phase** | **Middle exp.phase** | **Late exp. phase** | **Stationary phase** | **Early exp. phase** | **Middle exp. phase** | **Late exp. phase** | | **Stationary phase** | |
| Bromofuranone | | | | | | | | | | |
| 0 | 16.58±2.12 | 6.91±0.19 | 1.94±0.05 | 1.15± 0.03 | 15.47±2.19 | 8.83±0.47 | 1.94±0.07 | | 1.24±0.05 | |
| 40 µM | 11.21±1.10 | 5.61±0.25 | 1.85±0.07 | 1.11±0.03 | 15.89±1.58 | 7.99± 0.05 | 1.86±0.07 | | 1.18±0.05 | |
| 80 µM | 12.92±3.18 | 4.35±0.41 | 1.44±0.05 | 0.90±0.02 | 11.15± 1.62 | 7.52±0.55 | 1.77±0.06 | | 1.08±0.038 | |
| 120 µM | 11.88±3.07 | 3.61±0.74 | 1.38±0.03 | 0.89±0.02 | 11.18±1.62 | 7.10±4.56 | 1.73±0.10 | | 1.07±0.04 | |
| 160 µM | 10.30±1.74 | 3.93±0.53 | 1.32±0.02 | 0.88±0.03 | 9.82±2.28 | 6.35±0.39 | 1.64±0.05 | | 1.06±0.04 | |
| 3-oxo-N | | | | | | | | | | |
| 0 | 16.58±2.12 | 6.91±0.19 | 1.94±0.05 | 1.15± 0.03 | 15.47±2.19 | 8.83±0.47 | 1.94±0.07 | | 1.24±0.05 | |
| 20 µM | 14.49±1.45 | 6.48±0.30 | 1.92±0.05 | 1.10±0.02 | 13.64±2.11 | 8.56±0.48 | 1.85±0.08 | | 1.13±0.02 | |
| 40 µM | 14.78±1.90 | 5.49±0.41 | 1.69±0.07 | 1.08±0.03 | 14.20± 0.92 | 8.13± 0.45 | 1.80±0.08 | | 1.13± 0.06 | |
| 80 µM | 10.13±1.04 | 4.91±0.17 | 1.55±0.06 | 0.97±0.05 | 13.55±1.63 | 7.05±0.48 | 1.66±0.07 | | 1.03±0.05 | |
| 120 µM | 11.07±2.58 | 4.79±0.39 | 1.52±0.06 | 0.91±0.04 | 11.23±1.97 | 6.64±0.48 | 1.53±0.09 | | 1.00±0.04 | |
| 160 µM | 9.65±0.73 | 4.89±0.35 | 1.43±0.08 | 0.89±0.02 | 6.43±2.60 | 4.22±0.46 | 1.19±0.06 | | 0.78±0.04 | |
| γ-aminobutyric acid | | | | | | | | | | |
| 0 | 16.58±2.12 | 6.91±0.19 | 1.94±0.05 | 1.15± 0.03 | 15.47±2.19 | 8.83±0.47 | | 1.94±0.07 | | 1.24±0.05 |
| 1 mM | 15.10±1.00 | 6.28±0.20 | 1.79±0.03 | 1.05±0.009 | 13.78±2.00 | 8.63±0.05 | | 1.90±0.07 | | 1.14±0.02 |
| 2 mM | 13.55±1.29 | 5.72±0.41 | 1.80±0.06 | 1.05±0.03 | 13.18±1.64 | 8.41±0.52 | | 1.78±0.08 | | 1.08±0.05 |
| 5 mM | 11.24±3.01 | 4.78±0.38 | 1.50±0.05 | 0.97±0.01 | 13.02±1.68 | 8.08±0.61 | | 1.74±0.10 | | 1.07± 0.04 |
| 10 mM | 11.42±0.78 | 5.08±0.45 | 1.68±0.06 | 1.00±0.03 | 13.52±1.65 | 7.67±0.54 | | 1.69±0.06 | | 1.04±0.04 |
| 20 mM | 9.36±1.40 | 4.35±0.04 | 1.54±0.06 | 0.95±0.02 | 13.92±1.65 | 7.25±0.58 | | 1.63±0.07 | | 1.00±0.01 |
| 50 mM | 10.67±0.98 | 4.04±0.02 | 1.43±0.07 | 0.90±0.03 | 9.14±1.97 | 5.84±0.56 | | 1.31±0.09 | | 0.94±0.04 |

**Supplemental Table S4.** Specific AHL production rate exhibited by QSI-treated *D. vulgaris* and *Db. corrodens* planktonic cells in saline medium during early, mid, late exponential phases and stationary phase. No QSI was added to control.

| QSI concentration | Specific AHL production rate exhibited by QSI-treated *D. vulgaris* (×10^-12^ nmoles/cells/h) | | | | Specific AHL production rate exhibited by QSI-treated *Db. corrodens* (×10^-12^ nmoles/cells/h) | | | |
| --- | --- | --- | --- | --- | --- | --- | --- | --- |
|  | **Early exp. phase** | **Middle exp. phase** | **Late exp. phase** | **Stationary phase** | **Early exp. phase** | **Middle exp. phase** | **Late exp. phase** | **Stationary phase** |
| Bromofuranone | | | | | | | | |
| 0 | 23.20±4.26 | 10.21±1.97 | 1.63±0.27 | 0.93±0.06 | 30.61±4.84 | 7.14±0.86 | 1.76±0.68 | 0.86±0.28 |
| 40 µM | 20.92±1.71 | 8.52±0.90 | 0.69±0.15 | 0.28±0.09 | 27.33±8.37 | 6.95±1.08 | 0.98±0.22 | 0.62±0.07 |
| 80 µM | 23.50±3.08 | 5.67±0.71 | 0.43±0.16 | 0.22±0.03 | 28.08±8.95 | 7.14±1.17 | 7.77±0.21 | 0.24±0.09 |
| 120 µM | 23.55±3.11 | 4.80±0.74 | 0.45±0.06 | 0.18±0.04 | 19.73±3.03 | 6.93±2.46 | 0.67±0.14 | 0.19±0.09 |
| 160 µM | 22.68±3.31 | 3.45±0.46 | 0.41±0.07 | 0.17±0.04 | 19.98±4.31 | 5.61±1.53 | 0.48±0.23 | 0.15±0.08 |
| 3-oxo-N | | | | | | | | |
| 0 | 23.20±4.26 | 10.21±1.97 | 1.63±0.27 | 0.93±0.06 | 30.61±4.84 | 7.14±0.86 | 1.76±0.68 | 0.86±0.28 |
| 20 µM | 26.4±1.29 | 5.91±0.53 | 0.80±0.08 | 0.27±0.02 | 27.26±6.55 | 7.89±1.53 | 1.37±0.37 | 0.58±0.22 |
| 40 µM | 21.86±2.29 | 6.05±0.37 | 0.54±0.08 | 0.23±0.07 | 19.65±3.85 | 6.77±3.45 | 0.82±0.44 | 0.25±0.12 |
| 80 µM | 24.92±1.19 | 4.79±0.73 | 0.40±0.08 | 0.20 ±0.05 | 19.97±4.44 | 6.83±3.51 | 0.96±0.30 | 0.34±0.05 |
| 120 µM | 24.86±3.59 | 3.83±0.68 | 0.49±0.16 | 0.14±0.09 | 17.11±4.64 | 4.15±1.20 | 7.20±0.16 | 0.25±0.08 |
| 160 µM | 23.20±2.95 | 3.39±0.63 | 0.31±0.13 | 0.14±0.05 | 22.30±0.57 | 5.97±1.85 | 4.00±0.20 | 0.24±0.01 |
| γ-aminobutyric acid | | | | | | | | |
| 0 | 23.20±4.26 | 10.21±1.97 | 1.63±0.27 | 0.93±0.06 | 30.61±4.84 | 7.14±0.86 | 1.76±0.68 | 0.86±0.28 |
| 1 mM | 25.68±2.43 | 9.04±0.55 | 1.14±0.22 | 0.49±0.06 | 29.54±4.05 | 11.20±2.51 | 1.40±0.30 | 0.55±0.16 |
| 2 mM | 24.63±0.49 | 7.28±1.27 | 0.88±0.08 | 0.39±0.08 | 28.92±9.41 | 7.37±1.22 | 1.03±0.18 | 0.48±0.12 |
| 5 mM | 19.11±8.12 | 3.04±0.88 | 0.71±0.13 | 0.34±0.12 | 27.02±8.25 | 8.30±3.47 | 0.82±0.24 | 0.44±0.08 |
| 10 mM | 17.96±0.43 | 4.67±0.88 | 0.36±0.03 | 0.27±0.07 | 17.30±4.53 | 6.64±2.56 | 0.73±0.30 | 0.35±0.12 |
| 20 mM | 16.31±3.08 | 4.22±0.99 | 0.54±0.21 | 0.13±0.02 | 13.53±0.50 | 5.84±1.65 | 0.31±0.02 | 0.43±0.09 |
| 50 mM | 22.02±3.82 | 1.05±0.11 | 0.48±0.21 | 0.07±0.01 | 16.76±9.15 | 4.16±2.28 | 0.53±0.16 | 0.23±0.09 |


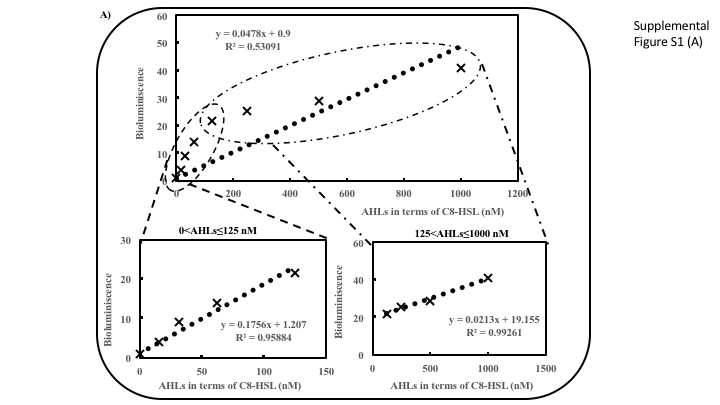


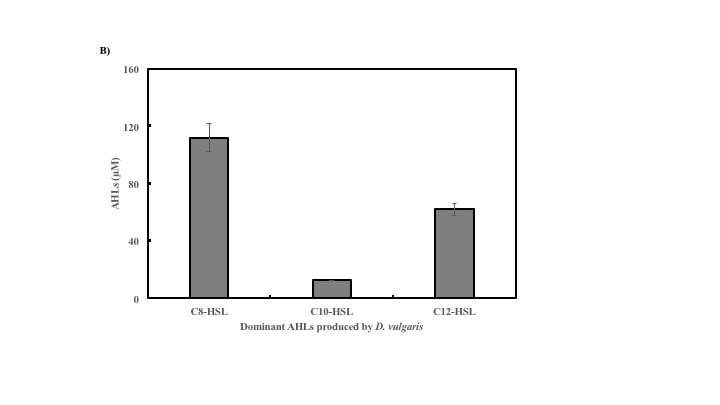


**Supplemental Figure S1**. Total AHLs produced by *D. vulgaris* and *Db. corrodens* were quantitavely estimated using bioluminescence assay with *Agrobacterium tumifaciens* NT1 biosensor. **(A)** AHL-bioluminescence standard curve for quantifying total AHLs produced, derived using C8-HSL. C8-HSL was identified as the dominant AHL produced by *D. vulgaris* and *Db. corrodens* using LC-MS/MS. Two best-fit curves covering two specific linear ranges were resolved from the standard curve. **(B)** Dominant AHLs produced by SRB, resolved and detected using LC-MS/MS.

**
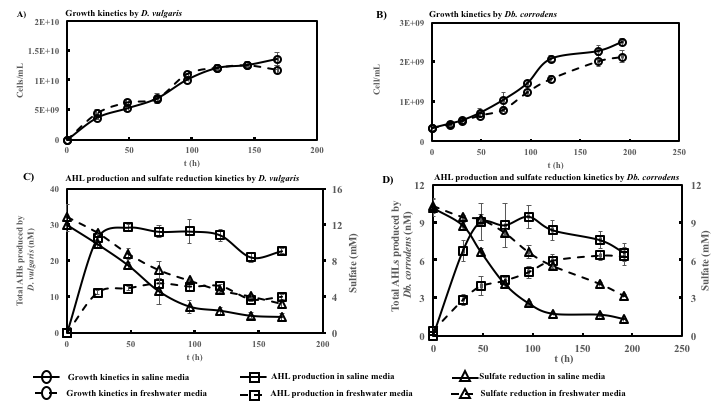
**

**Supplemental Figure S2**. Salinity enhanced kinetics involving sulfate reduction and AHL production by *D. vulgaris* and *Db. corrodens*. **(A)** Growth kinetics by *D. vulgaris* in saline and freshwater media. **(B)** Growth kinetics by *Db. corrodens* in saline and freshwater media.**(C)** Sulfate reduction and AHL production kinetics by *D. vulgaris* in saline and freshwater media. **(D)** Sulfate reduction and AHL production kinetics by *Db. corrodens* in saline and freshwater media. Results are presented as mean ± standard deviation (n=3).


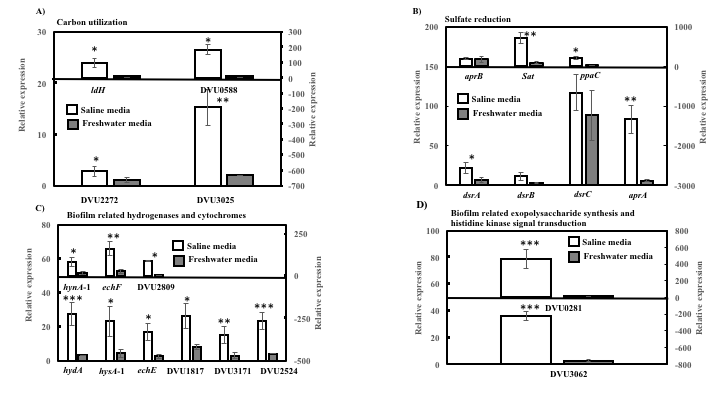


**Supplemental Figure S3**. RT-qPCR analysis of relative expression of selected genes related to carbon metabolism, sulfate reduction, electron transfer and biofilm formation in *D. vulgaris* planktonic cells under saline and freshwater conditions**. (A)** Relative expression of carbon metabolism enzymes pyruvate formate lyase DVU2272 and pyruvate dehydrogenase DVU3025 in the primary left y-axis, and lactate dehydrogenase *ldh*, formate dehydrogenase DUV0588 in the secondary right y-axis. **(B)** Relative expression of dissimilatory sulfite reductase *dsrA*, *dsrB*, *dsrC* and adenosine 5’-phosphosulfate reductase alpha subunit *aprA* in the primary left y-axis, and adenosine 5’-phosphosulfate reductase beta subunit *aprB,* sulfate adenylytransferase *Sat,* pyrophosphatase *ppaC* in the secondary right y-axis. **(C)** Relative expression of Fe hydrogenase *hydA*, NiFeSe hydrogenase *hysA*-1, Ech hydrogenases *echE*, formate dehydrogenase DVU1817and *c*3-type cytochromes DVU3171 and DVU2524 in the primary left y-axis, as well as NiFe hydrogenase *hynA*-1, Ech hydrogenase *echF*, and *c*3-type cytochrome DVU2809 in the secondary right y-axis. **(D)** Relative expression of sensor histidine kinase response regulator DVU3062 in the primary left y-axis and exopolysaccharide synthesis protein DVU0281 in the secondary right y-axis. Relative expression refers to the transcript level of a specific gene normalized with that of reference gene *recA*. Results are presented as mean ± standard deviation (n=3). Significant difference (p<0.05) indicated by (*); (p<0.01) indicated by (**) and (p<0.001) indicated by {***).

**REFERENCE**

Clark, M.E., Edelmann, R.E., Duley, M.L., Wall, J.D., and Fields, M.W. (2007). Biofilm formation in *Desulfovibrio vulgaris* Hildenborough is dependent upon protein filaments. *Environ. Microbiol.* 9**,** 2844-2854.

Clark, M.E., He, Z., Redding, A.M., Joachimiak, M.P., Keasling, J.D., Zhou, J.Z., Arkin, A.P., Mukhopadhyay, A., and Fields, M.W. (2012). Transcriptomic and proteomic analyses of *Desulfovibrio vulgaris* biofilms: carbon and energy flow contribute to the distinct biofilm growth state. *BMC genomics* 13**,** 138.

Heidelberg, J.F., Seshadri, R., Haveman, S.A., Hemme, C.L., Paulsen, I.T., Kolonay, J.F., Eisen, J.A., Ward, N., Methe, B., and Brinkac, L.M. (2004). The genome sequence of the anaerobic, sulfate-reducing bacterium *Desulfovibrio vulgaris* Hildenborough. *Nat. Biotechnol.* 22**,** 554.

Keller, K., and Wall, J. (2011a). Genetics and Molecular Biology of the Electron Flow for Sulfate Respiration in *Desulfovibrio*. *Front. Microbiol.* 2.

Keller, K.L., and Wall, J.D. (2011b). Genetics and molecular biology of the electron flow for sulfate respiration in *Desulfovibrio*. *Front. Microbiol.* 2**,** 135.

Mcinerney, M.J., and Bryant, M.P. (1981). Anaerobic degradation of lactate by syntrophic associations of *Methanosarcina barkeri* and *Desulfovibrio species* and effect of H2 on acetate degradation. *Appl. Environ. Microbiol.* 41**,** 346-354.

Meyer, B., Kuehl, J.V., Price, M.N., Ray, J., Deutschbauer, A.M., Arkin, A.P., and Stahl, D.A. (2014). The energy‐conserving electron transfer system used by Desulfovibrio alaskensis strain G20 during pyruvate fermentation involves reduction of endogenously formed fumarate and cytoplasmic and membrane‐bound complexes, Hdr‐Flox and Rnf. *Environ. Microbiol.* 16**,** 3463-3486.

Pereira, P.M., He, Q., Valente, F.M., Xavier, A.V., Zhou, J., Pereira, I.A., and Louro, R.O. (2008). Energy metabolism in Desulfovibrio vulgaris Hildenborough: insights from transcriptome analysis. *Antonie Van Leeuwenhoek* 93**,** 347-362.

Qi, Z., Chen, L., and Zhang, W. (2016). Comparison of transcriptional heterogeneity of eight genes between batch Desulfovibrio vulgaris biofilm and planktonic culture at a single-cell level. *Front. Microbiol.* 7**,** 597.

Qi, Z., Pei, G., Chen, L., and Zhang, W. (2014). Single-cell analysis reveals gene-expression heterogeneity in syntrophic dual-culture of Desulfovibrio vulgaris with Methanosarcina barkeri. *Sci. Rep.* 4**,** 7478.

Rajeev, L., Luning, E.G., Dehal, P.S., Price, M.N., Arkin, A.P., and Mukhopadhyay, A. (2011). Systematic mapping of two component response regulators to gene targets in a model sulfate reducing bacterium. *Genome biology* 12**,** R99.

Scarascia, G., Wang, T., and Hong, P.-Y. (2016). Quorum Sensing and the Use of Quorum Quenchers as Natural Biocides to Inhibit Sulfate-Reducing Bacteria. *Antibiotics* 5**,** 39.

Slater, H., Alvarez‐Morales, A., Barber, C.E., Daniels, M.J., and Dow, J.M. (2000). A two‐component system involving an HD‐GYP domain protein links cell–cell signalling to pathogenicity gene expression in Xanthomonas campestris. *Mol. Microbiol.* 38**,** 986-1003.

Takeda, S.I., Fujisawa, Y., Matsubara, M., Aiba, H., and Mizuno, T. (2001). A novel feature of the multistep phosphorelay in Escherichia coli: a revised model of the RcsC→ YojN→ RcsB signalling pathway implicated in capsular synthesis and swarming behaviour. *Mol. Microbiol.* 40**,** 440-450.

Voordouw, G., Niviere, V., Ferris, F.G., Fedorak, P.M., and Westlake, D.W. (1990). Distribution of hydrogenase genes in Desulfovibrio spp. and their use in identification of species from the oil field environment. *Appl. Environ. Microbiol.* 56**,** 3748-3754.

Zhang, W., Culley, D.E., Nie, L., and Scholten, J.C. (2007). Comparative transcriptome analysis of Desulfovibrio vulgaris grown in planktonic culture and mature biofilm on a steel surface. *Appl. Environ. Microbiol.* 76**,** 447-457.

Zhang, W., Culley, D.E., Wu, G., and Brockman, F.J. (2006). Two-component signal transduction systems of Desulfovibrio vulgaris: structural and phylogenetic analysis and deduction of putative cognate pairs. *J Mol. Evol.* 62**,** 473-487.
